# Supplementary figures and images for: Optimizing multi-environment trials in the Southern US Rice belt via smart-climate-soil prediction-based models and economic importance
Source: Front Plant Sci. 2024 Oct 23;15:1458701. doi: 10.3389/fpls.2024.1458701 (PMC11537932; doi:10.3389/fpls.2024.1458701)

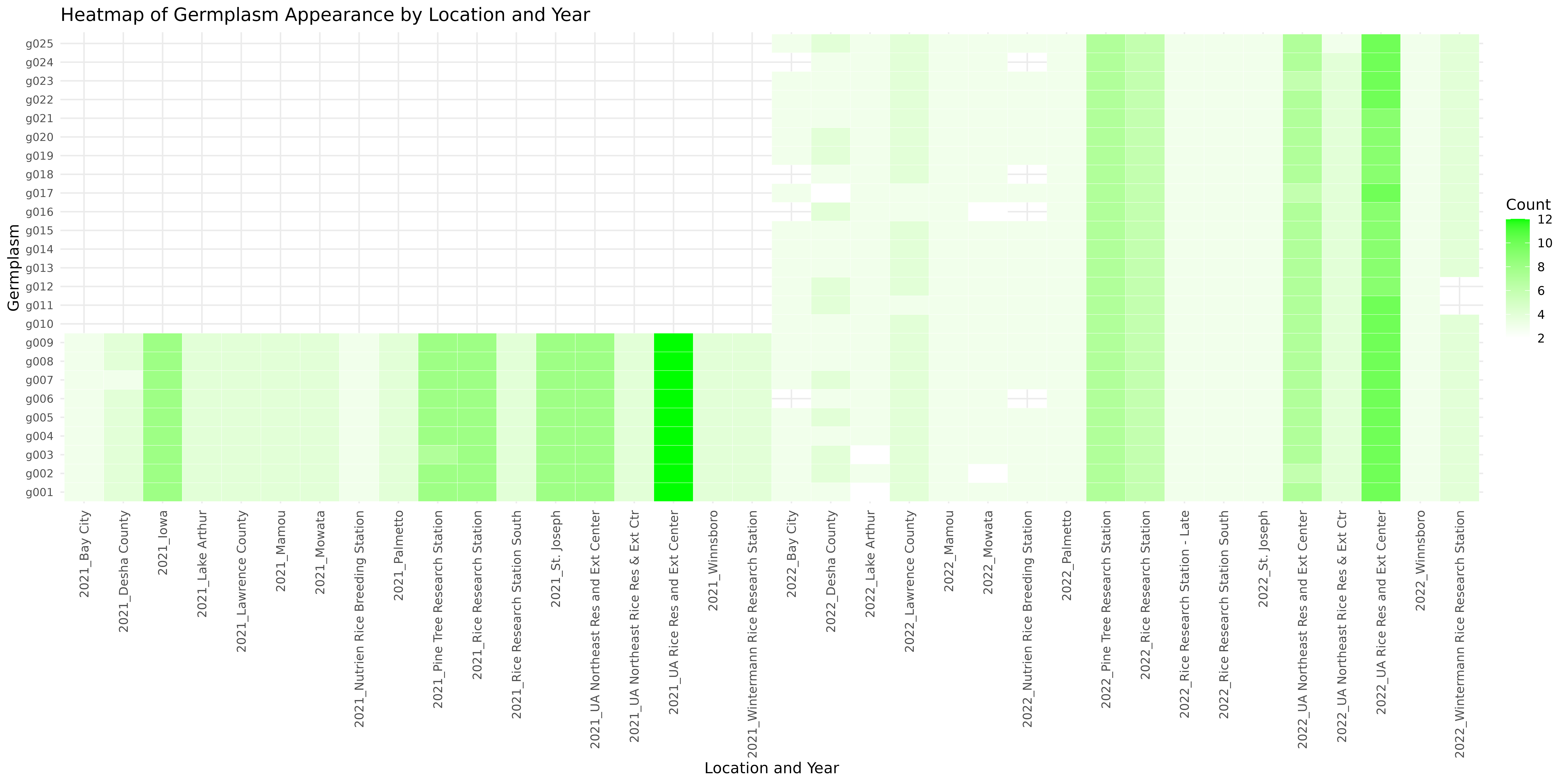

Supplement: Supplementary Figure 1 — Number of phenotypes evaluated per year and location for each of the 25 genotypes included in LSU’s advanced trials. [file Image1.jpeg]

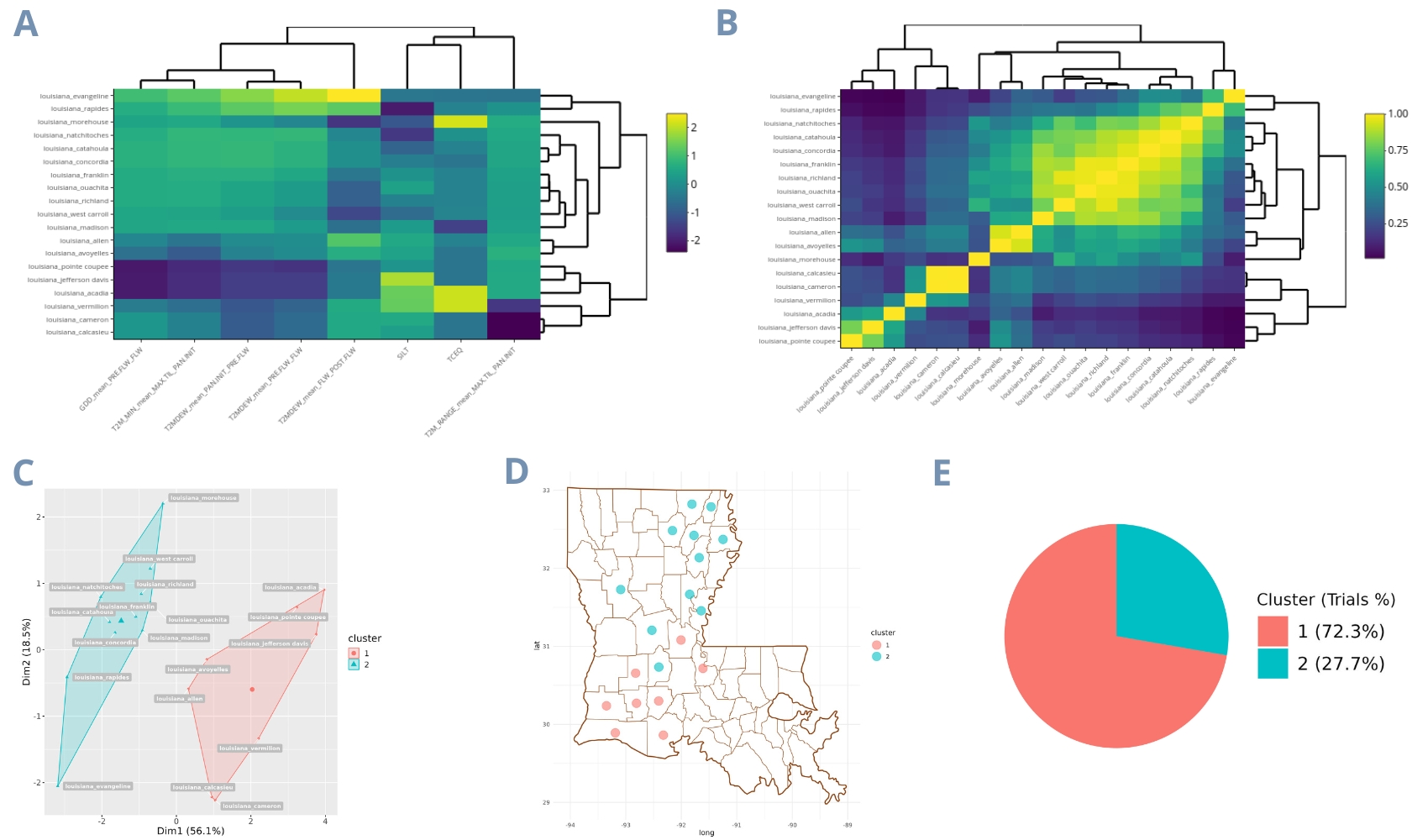

Supplement: Supplementary Figure 2 — Clustering and characterization of Louisiana TPEs using the environmental covariates matrix. (A) The environmental covariates matrix; (B) Environmental relationship matrix; (C) Clusters defining the dataset mega-environments; (D) All locations separated by cluster on the map; (E) Trials percentage in each cluster. The colors of each cluster are the same in all images. [file Image2.jpeg]
